# Deciphering the Microbiome-Gut-Eye Axis: A Mendelian Randomization Analysis of the Causal Influence of Gut Microbiota on Myopia

**DOI:** 10.2174/0113862073385717250415110224

**Published:** 2025-04-29

**Authors:** Weicheng Xu, Wei Shi

**Affiliations:** 1 First Clinical Medical College, Nanjing University of Chinese Medicine, Nanjing, Jiangsu, China;; 2 Department of Ophthalmology, Rugao Affiliated Hospital of Nanjing University of Chinese Medicine, Nantong, Jiangsu, China;; 3 Department of Ophthalmology, Jiangsu Province Hospital of Chinese Medicine, Nanjing, Jiangsu, China

**Keywords:** Myopia, gut microbiota, mendelian randomization, ocular health, microbiome-gut-eye axis, GWAS

## Abstract

**Introduction:**

The intricate relationship between the gut microbiome and myopia is increasingly recognized, underscoring the need to explore its causal dynamics. Despite emerging evidence, the influence of Gut Microbiota (GM) on ocular development remains underexplored.

**Methods:**

This study utilized Mendelian Randomization (MR) to investigate the causal impact of GM on the development of myopia. Instrumental variables (IVs) were identified from Genome-Wide Association Studies (GWAS), focusing on genetic variants significantly associated with microbiome composition. A comprehensive array of MR techniques was applied to ensure a robust estimation of causal effects and to adjust for potential confounders and pleiotropy.

**Results:**

The Inverse-Variance Weighted (IVW) method was used to identify significant associations between GM and myopia. Increased risk of myopia was linked to the class *Betaproteobacteria* (OR=1.01, 95% CI 1.004-1.017, *P*=0.003), the order *Burkholderiales* (OR=1.009, 95% CI 1.001-1.016, *P*=0.02), the family *Oxalobacteraceae* (OR=1.005, 95% CI 1.001-1.01, *P*=0.023), and several genera including *Eubacterium xylanophilum group* (OR=1.007, 95% CI 1.001-1.013, *P*=0.033), and *Bifidobacterium* (OR=1.005, 95% CI 1-1.01, *P*=0.038). Protective effects were noted for the order *Mollicutes RF9* (OR=0.994, 95% CI 0.99-0.999, *P*=0.014), the genus *Allisonella* (OR=0.996, 95% CI 0.993-0.999, *P*=0.019), the genus *Lachnospiraceae UCG001* (OR=0.994, 95% CI 0.989-1, *P*=0.045), and the family *Enterobacteraceae* (OR=0.991, 95% CI 0.982-1, *P*=0.047) and order *Enterobacteriales* (OR=0.991, 95% CI 0.982-1, *P*=0.047). Sensitivity analyses further confirmed the robustness of these findings.

**Discussion:**

This study provides causal evidence for the "Microbiome-Gut-Eye Axis" in myopia development, identifying specific gut microbiota that influence myopia risk. These findings suggest potential for microbiota-targeted interventions, warranting further research in diverse populations.

**Conclusions:**

The findings support the "Microbiome-Gut-Eye Axis" as a potential factor in myopia pathogenesis and highlight microbiota-targeted interventions as novel therapeutic strategies for managing myopia. This study lays the groundwork for further research on how modifying GM can influence eye health and offers new perspectives on preventive health strategies.

## 1 INTRODUCTION

Myopia, or nearsightedness, is a prevalent refractive error and a major cause of vision decline in adolescents and adults. It significantly affects personal quality of life and imposes a substantial burden on society [1]. High myopia, in particular, is associated with eyeball elongation, leading to pathological changes in the choroid and retina. These changes significantly increase the risk of cataracts, glaucoma, macular degeneration, and retinal detachment, potentially leading to irreversible visual impairment and blindness. Currently, approximately 2 billion people worldwide suffer from myopia, accounting for 28.3% of the global population. Among them, around 277 million people have high myopia, representing 4.0% of the global population. Projections indicate that by 2050, myopia cases will rise to 4.76 billion, affecting 49.8% of the global population. Nearly 1 billion individuals will suffer from high myopia, accounting for 9.8% of the global population. Consequently, the prevention and control of myopia have become critical public health challenges [2].

Microorganisms, such as viruses, bacteria, fungi, and protozoa, form a vast group of biological entities abundantly present in the human gastrointestinal tract. The gut harbors over 1,000 species of microbes, with major groups including Bacteroidetes, Actinobacteria, Firmicutes, and Proteobacteria. These GM are closely linked to various human diseases. The surface area of the human gastrointestinal tract is approximately 250 to 400 square meters. Changes in GM composition can affect gut function and modulate immune, metabolic, and inflammatory pathways in the body [3]. This modulation affects various target organs, including the brain, bones, skin, heart, breast, lungs, colon, rectum, prostate, stomach, endometrium, and ovaries. Research on these effects includes both theoretical exploration and extensive scientific experimentation [4-9].

The intricate relationships between the microbiome and various physiological systems have become a central focus of scientific research. In this context, the concept of the "Microbiome-Gut-Eye Axis" has emerged. This concept highlights the interconnections between GM, the gut, and the eyes. Extensive research has confirmed these links, revealing that eye diseases like uveitis, glaucoma, diabetic retinopathy, Age-related Macular Degeneration (AMD), Retinal Artery Occlusion (RAO), and Central Serous Chorioretinopathy (CSCR) are closely associated with GM [10-15].

MR is an experimental design method named after the geneticist Gregor Mendel. This method uses genetic variations to assess the relationship between genetic abnormalities and disease risk or other health outcomes. A key advantage of MR is that genetic variations are determined at conception and are randomly distributed among individuals. This random distribution helps minimize confounding factors, thereby enhancing the accuracy of causal inferences. This approach allows researchers to more reliably uncover true relationships between risk factors and health outcomes [16].

This study used large-scale public GWAS data to conduct a two-sample MR analysis. Genetic variants associated with GM composition were used as IVs to investigate the potential causal effects of 211 GM species on myopia. From an MR theoretical perspective, this research validated the "Microbiome-Gut-Eye Axis" concept. The goal was to explore the role of GM regulation in reducing the incidence and progression of myopia, offering a new therapeutic approach for myopia prevention and control.

## 2 MATERIALS AND METHODS

### 2.1 Study Design and Data Sources

This MR study aimed to clarify the causal relationships between GM and myopia. We used data from GWAS that identified host genetic factors influencing human gut microbiome composition, as reported by Kurilshikov *et al.* in 2021 in *Nature Genetics* [17]. The myopia dataset (GWAS ID: ukb-b-6353) was sourced from the MRC Integrative Epidemiology Unit (IEU) at the University of Bristol. This dataset includes information on individuals requiring vision correction primarily for distance, categorized as myopia. The 'ukb-b-6353' dataset comprises 460,536 participants, including 37,362 cases and 423,174 controls. Fig (**1**) illustrates the flowchart detailing the principles of the MR study linking GM taxa with myopia. We followed the STROBE-MR checklist to improve the reporting of MR studies, as recommended in the literature [18].

### 2.2 Selection of IVs

In the genome-wide analysis of GM, we used a threshold of *P*<1×10^-5 for selecting SNPs instead of the conventional *P*<5×10^-8. This choice was made because the stricter threshold of *P*<5×10^-8 did not yield enough SNPs to serve as IVs for analysis. The threshold of *P*<1×10^-5 is also adequate for MR analysis, as shown in previous studies [19, 20]. Additionally, we conducted a Linkage Disequilibrium (LD) analysis on the selected SNPs, using a criterion of R^2 <0.001 and a clumping distance of 10,000 kb. To prevent uncertainties in allele matching from affecting the causal assessment between GM and myopia, we excluded palindromic SNPs [21].

### 2.3 Statistical Analysis

In this study, all statistical analyses were performed using version 4.4.0 of the R software. The 'TwoSampleMR' package in R was used to conduct MR analysis, aiming to investigate the causal relationship between GM and myopia. Our MR analysis methodology varied based on the number of SNPs identified for each bacterial taxon. For taxa associated with a single SNP, we used a Wald ratio test to estimate the causal effect. When two SNPs were identified, we limited the analysis to the Inverse Variance Weighted (IVW) method. For taxa linked to more than two SNPs, we used a broader range of MR techniques, including MR-Egger, Weighted median, IVW, Simple mode, and Weighted mode, to provide a comprehensive evaluation of causal effects [19, 22-24].

The IVW method is crucial in MR, using genetic variants as IVs to deduce causal relationships. This method enhances the precision of causal inferences by weighting each variant inversely to its variance. To ensure the rigor of our findings, we meticulously assessed and weighted these variants, reinforcing the validity of our conclusion through sensitivity analyses aimed at detecting potential biases. Additionally, we adhered to a stringent significance threshold of *P*<0.05 throughout the study [21, 25].

### 2.4 Sensitivity Analyses

To ensure the robustness of our findings, we conducted multiple sensitivity analyses. These included Cochran’s Q test to assess heterogeneity among genetic instruments and MR-Egger regression to check for directional pleiotropy. Additionally, we used funnel plots and leave-one-out analyses to identify influential outliers and confirm the stability of our results [26].

## 3 RESULTS

### 3.1 Overview of IVs in Taxa

In our study, we identified 2,329 SNPs associated with 211 bacterial taxa, all reaching statistical significance at *P*<1×10^-5. These SNPs were distributed across various taxonomic levels: 108 in 9 phyla, 188 in 16 classes, 230 in 20 orders, 401 in 35 families, and 1,402 in 131 genera. Notably, IVs were identified for all taxa except the family *Christensenellaceae* (id.1866). This comprehensive set of SNPs provides a robust foundation for our MR analysis, exploring the causal effects of GM on myopia.

### 3.2 Associations of Gut Bacterial Taxa with Myopia

Fig. (**2**) displays the preliminary results from the analysis investigating the associations between genetically proxied gut bacterial taxa and the risk of myopia. Notably, taxa that could not be analyzed using all five methods were excluded. These included genus *Blautia* (id.1992; IVW *P*=0.799), genus *Erysipelotrichaceae UCG003* (id.11384; Wald ratio *P*=0.956), and genus *Lachnospira* (id.2004; Wald ratio *P*=0.050), which showed no significant association with myopia. Among the 211 bacterial taxa examined, 13 were found to exhibit a causal association with myopia, as illustrated in Fig. (**3**).

Our analysis revealed significant associations between specific GM and the risk of developing myopia (**S1** Fig A, F-J). For instance, the class *Betaproteobacteria* (id.2867) showed a considerable increase in myopia risk (IVW OR=1.01, 95% CI 1.004-1.017, *P*=0.003). Similarly, the order *Burkholderiales* (id.2874) (IVW OR=1.009, 95% CI 1.001-1.016, *P*=0.02), family *Oxalobacteraceae* (id.2966) (IVW OR=1.005, 95% CI 1.001-1.01, *P*=0.023), and several genera including an unknown genus (id.2755) (IVW OR=1.005, 95% CI 1.001-1.009, *P*=0.026), *Eubacterium xylanophilum group* (id.14375) (IVW OR=1.007, 95% CI 1.001-1.013, *P*=0.033), and *Bifidobacterium* (id.436) (IVW OR=1.005, 95% CI 1-1.01, *P*=0.038) also exhibited increases in myopia risk.

Conversely, certain taxa demonstrated protective effects against myopia (**S1** Fig B-E, K-M), such as the order *Mollicutes RF9* (id.11579) (IVW OR=0.994, 95% CI 0.99-0.999, *P*=0.014). Additional taxa showing similar protective effects included an unknown family (id.1000005471) and genus (id.1000005472) (both with IVW OR=0.994, 95% CI 0.99-0.999, *P*=0.014), genus *Allisonella* (id.2174) (IVW OR=0.996, 95% CI 0.993-0.999, *P*=0.019), genus *Lachnospiraceae* *UCG001* (id.11321) (IVW OR=0.994, 95% CI 0.989-1, *P*=0.045), and the family *Enterobacteriaceae* and order *Enterobacteriales* (both with IVW OR=0.991, 95% CI 0.982-1, *P*=0.047).

### 3.3 Sensitivity Analyses and Pleiotropy Detection

Comprehensive sensitivity analyses were performed, including Cochran’s Q tests for heterogeneity and MR-Egger intercept tests to check for pleiotropy. Significant heterogeneity was observed for the family *Oxalobacteraceae* (id.2966) (MR Egger *P*=0.0098, IVW *P*=0.0132). However, no significant heterogeneity or pleiotropy was detected across the other taxa associated with myopia, affirming the robustness of our findings. Detailed results of these analyses are provided in Table **1**, and visual evidence from funnel plots is shown in Fig. **S2**. No indication of horizontal pleiotropy was detected among the 13 taxa associated with myopia (*P*>0.05), further validating the stability of the MR findings detailed in Fig. **S3**.

Overall, our MR study presents compelling evidence that specific GM taxa have significant and differential impacts on the risk of myopia. This nuanced understanding offers potential targets for preventing or managing myopia through GM modulation.

## 4 DISCUSSION

Research indicates that the gut microbiome affects the development and progression of various eye diseases through several mechanisms. Firstly, it plays a critical role in maintaining immune system balance and regulating inflammatory responses. Microbial metabolites, such as short-chain fatty acids, help modulate immune cells and preserve the integrity of the gut barrier. Secondly, dysbiosis, or microbial imbalance, can lead to systemic inflammation. Microbes and their metabolites can travel through the bloodstream to the eyes, potentially triggering eye diseases. Additionally, specific alterations in the gut microbiome are linked to the pathological features and genetic predispositions of these eye conditions [3].

Myopia, one of the most common clinical conditions in ophthalmology, significantly impacts people's lives and health, particularly in cases of high myopia. Investigating the GM associated with myopia, guided by the 'Microbiome-Gut-Eye Axis' theory, is of critical clinical relevance for understanding and managing this condition.

Li *et al.* [27] identified differences in the GM between myopic and non-myopic mice, notably with a decreased relative abundance of the phylum *Firmicutes* and an increased abundance of the phylum *Actinobacteria* in myopia. In our study, results for the phylum *Firmicutes* (IVW OR=0.998, 95% CI 0.991-1.004, *P*=0.473) and the phylum *Actinobacteria* (IVW OR=1.005, 95% CI 0.998-1.012, *P*=0.159) were consistent with the impact of GM on myopia as suggested by MR. However, they were not statistically significant (*P*>0.05). The observed discrepancies could be attributed to differences in microbial abundance between rodents and humans, as well as our primary sample population being European, which may explain the bias in microbial species observed.

In a study utilizing 16S rRNA gene sequencing, Omar *et al.* [28] analyzed fecal samples from 52 adults, comprising 23 nonmyopes, 8 progressive myopes (PM), and 21 stable myopes(SM). They discovered elevated levels of *Bifidobacterium*, *Bacteroides*, *Megamonas*, *Faecalibacterium*, *Coprococcus*, *Dorea*, *Roseburia*, and *Blautia* in the myopic groups (SM and PM) when compared to nonmyopes. Additionally, an increased abundance of bacteria involved in dopaminergic signaling regulation, such as *Clostridium*, *Ruminococcus*, *Bifidobacterium*, and *Bacteroides*, was noted. In particular, individuals with stable myopia showed a higher prevalence of *Prevotella copri* than those with progressive myopia. This aligns with findings from our research, which also demonstrated a significant presence of genus *Bifidobacterium* (id.436) (IVW OR=1.005, 95% CI 1-1.01, *P*=0.038) in the myopic population. Although no other overlapping GM were identified, possibly due to the limited sample size of 53 individuals, these findings underscore the relevance of studying GM in myopic populations.

Building on these findings, our study extends the body of evidence by demonstrating the causal associations of specific GM taxa with myopia. Sensitivity analyses further validated the robustness of our results, showing no significant heterogeneity or pleiotropy across most taxa associated with myopia. This strengthens the potential of targeting specific microbiota for therapeutic interventions. For instance, the protective effects observed in taxa, such as *Mollicutes RF9*, *Allisonella,* and *Lachnospiraceae UCG001,* suggest that enhancing or maintaining the abundance of these bacteria in the gut might contribute to preventing or mitigating myopia progression. Given the complexity and variability of GM interactions, future research should aim to explore these relationships in larger and more diverse populations, potentially leading to microbiota-based preventive strategies that could be personalized based on individual microbiome profiles.

## CONCLUSION

The integration of our results with existing literature underscores the potential of GM as both a biomarker and a therapeutic target for myopia. We advocate for a more nuanced approach to the clinical management of myopia, emphasizing the modulation of the microbiome as a promising strategy to prevent or slow its progression. Additional studies should further explore the underlying mechanisms and examine the therapeutic potential of microbiome-based interventions. These investigations could pave the way for personalized treatments for individuals at risk of myopia or its complications, shedding light on the deep connection within the microbiome-gut-eye axis. This holistic perspective could reshape the approach to myopia in both clinical and research settings, opening the door to innovative therapies that address the complex interactions between genetics, environment, and the microbiome.

## STUDY LIMITATIONS

However, there are some limitations in our study that should be acknowledged. We used a two-sample Mendelian randomization approach, with participants primarily of European descent. This focus on a specific demographic limits the ability to generalize the findings to other racial or ethnic groups. Therefore, caution is needed when applying these results to populations with different genetic backgrounds.

## Figures and Tables

**Fig. (1):**
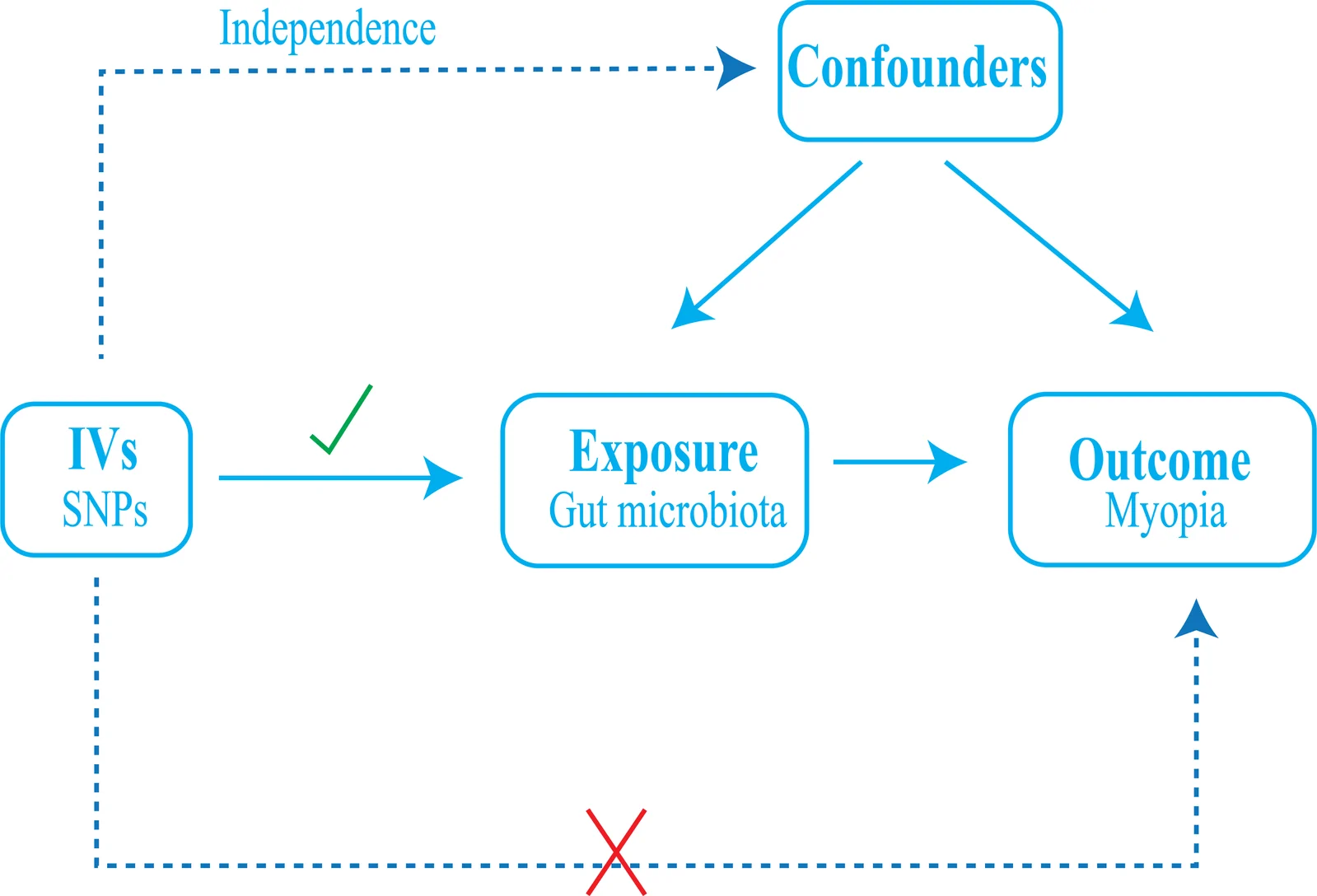
The principles of the MR study connecting GM taxa with myopia.

**Fig. (2):**
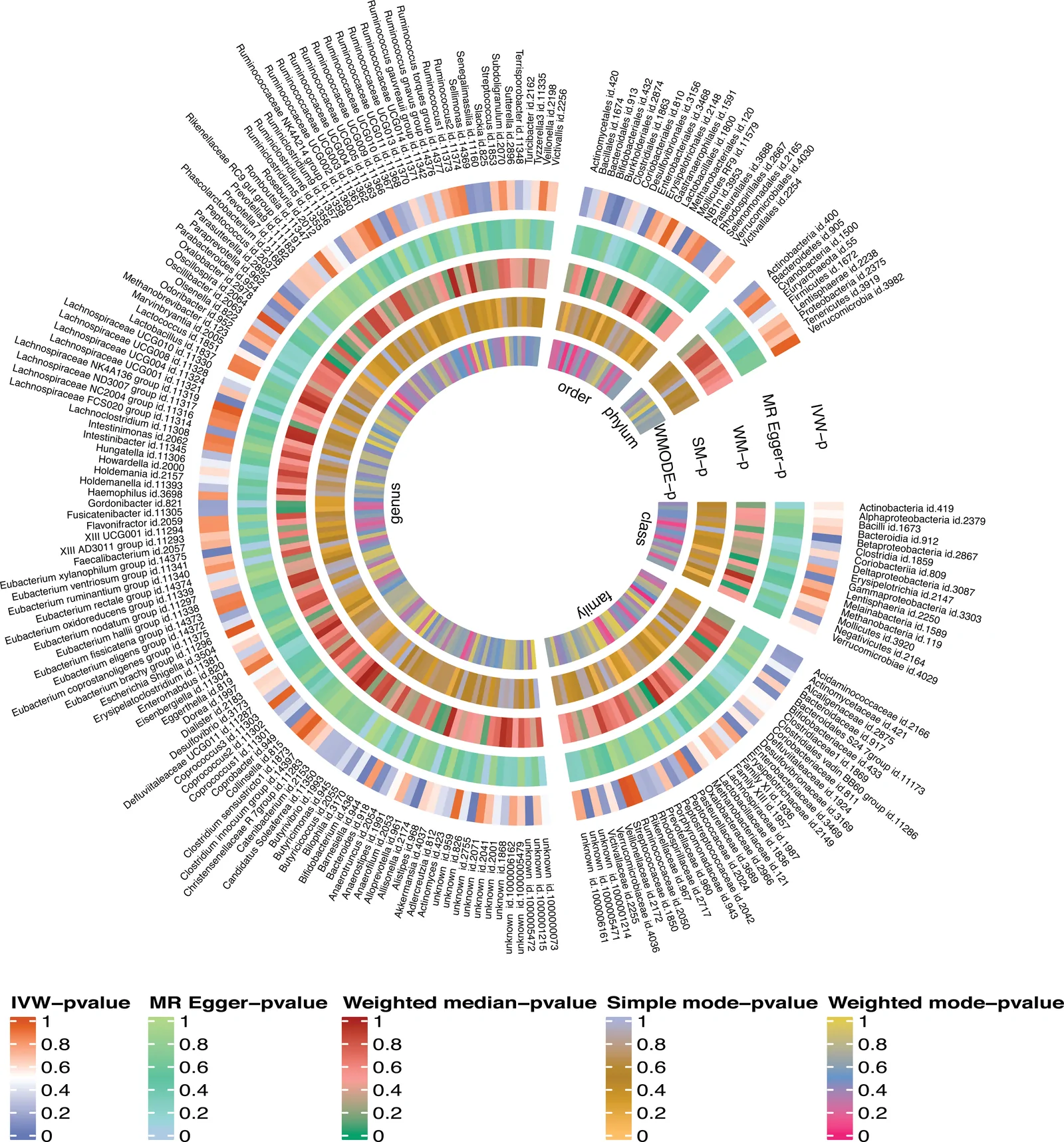
Results of the MR analysis between GM and Myopia analyzed using five different methods.

**Fig. (3):**
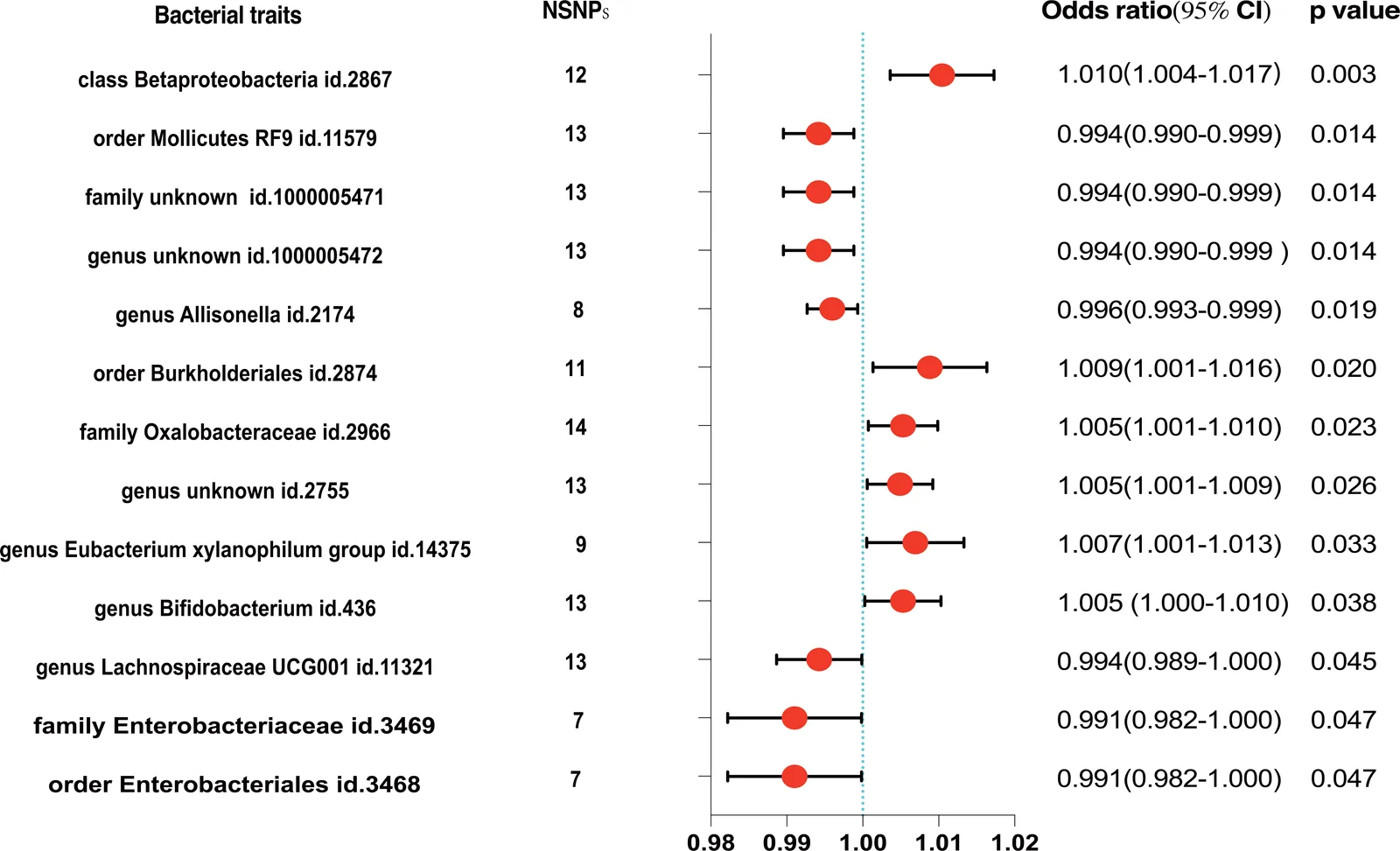
MR results of GM taxa with a causal relationship to myopia.

**Table 1:** Sensitivity analysis between GM and Myopia.

**Level**	**GM**	**Method**	**Q**	**Q-df**	***P***	**Intercept**	***P***
Class	*Betaproteobacteria* id.2867	MR Egger	12.574	10	0.248	0.000	0.733
		IVW	12.729	11	0.311		
Order	*Mollicutes RF9* id.11579	MR Egger	7.190	11	0.783	-0.001	0.058
		IVW	11.647	12	0.474		
Family	unknown id.1000005471	MR Egger	7.190	11	0.783	-0.001	0.058
		IVW	11.647	12	0.474		
Genus	unknown id.1000005472	MR Egger	7.190	11	0.783	-0.001	0.058
		IVW	11.647	12	0.474		
Genus	*Allisonella* id.2174	MR Egger	6.680	6	0.351	-0.001	0.551
		IVW	7.124	7	0.416		
Order	*Burkholderiales* id.2874	MR Egger	12.805	9	0.172	0.000	0.743
		IVW	12.969	10	0.225		
Family	*Oxalobacteraceae* id.2966	MR Egger	26.275	12	0.010	0.001	0.626
		IVW	26.823	13	0.013		
Genus	unknown id.2755	MR Egger	4.231	11	0.963	0.000	0.816
		IVW	4.288	12	0.978		
Genus	*Eubacterium xylanophilum group* id.14375	MR Egger	2.225	7	0.946	-0.002	0.052
		IVW	7.672	8	0.466		
Genus	*Bifidobacterium* id.436	MR Egger	7.381	11	0.767	0.000	0.347
		IVW	8.345	12	0.758		
Genus	*Lachnospiraceae UCG001* id.11321	MR Egger	15.128	11	0.177	0.002	0.170
		IVW	18.092	12	0.113		
Family	*Enterobacteriaceae* id.3469	MR Egger	7.989	5	0.157	0.001	0.676
		IVW	8.303	6	0.217		
Order	*Enterobacteriales* id.3468	MR Egger	7.989	5	0.157	0.001	0.676
		IVW	8.303	6	0.217		

## Data Availability

The authors would like to extend their sincere gratitude to the authors and contributors of all MiBioGen and GWAS studies whose summary statistics data were utilized.

## LIST OF ABBREVIATIONS

GM: Gut Microbiota
MR: Mendelian Randomization
GWAS: Genome-Wide Association Studies
IVW: Inverse-Variance Weighted

## STANDARDS OF REPORTING

STROBE-MR guidelines were followed.

## References

[1] Modjtahedi B.S., Abbott R.L., Fong D.S., Lum F., Tan D., Ang M., Chiarito S., Cotter S.A., Fernandez A.M., Grzybowski A., He M., Jacobs D.S., Jonas J.B., Kemper A., Lee K.A., Molinari A.D., Morgan I., Ohno-Matsui K., Repka M.X., Salim S., Wu P-C., Yao K., Zadnik K. (2021). Reducing the global burden of myopia by delaying the onset of myopia and reducing myopic progression in Children.. Ophthalmology.

[2] Jan C.L., Congdon N. (2018). Chinese national policy initiative for the management of childhood myopia.. Lancet Child Adolesc. Health.

[3] Zysset-Burri D.C., Morandi S., Herzog E.L., Berger L.E., Zinkernagel M.S. (2023). The role of the gut microbiome in eye diseases.. Prog. Retin. Eye Res..

[4] Liao W., Wei J., Liu C., Luo H., Ruan Y., Mai Y., Yu Q., Cao Z., Xu J., Zheng D., Sheng Z., Zhou X., Liu J. (2024). Magnesium-L-threonate treats Alzheimer’s disease by modulating the microbiota-gut-brain axis.. Neural Regen. Res..

[5] Huang K.C., Chuang P.Y., Yang T.Y., Tsai Y.H., Li Y.Y., Chang S.F. (2024). Diabetic rats induced using a high-fat diet and low-dose streptozotocin treatment exhibit gut microbiota dysbiosis and osteoporotic bone pathologies.. Nutrients.

[6] Mahmud M.R., Akter S., Tamanna S.K., Mazumder L., Esti I.Z., Banerjee S., Akter S., Hasan M.R., Acharjee M., Hossain M.S., Pirttilä A.M. (2022). Impact of gut microbiome on skin health: Gut-skin axis observed through the lenses of therapeutics and skin diseases.. Gut Microbes.

[7] Rahman M.M., Islam F. (2022). -Or-Rashid, M.H.; Mamun, A.A.; Rahaman, M.S.; Islam, M.M.; Meem, A.F.K.; Sutradhar, P.R.; Mitra, S.; Mimi, A.A.; Emran, T.B.; Fatimawali; Idroes, R.; Tallei, T.E.; Ahmed, M.; Cavalu, S. The gut microbiota (microbiome) in cardiovascular disease and its therapeutic regulation.. Front. Cell. Infect. Microbiol..

[8] Long Y., Tang L., Zhou Y., Zhao S., Zhu H. (2023). Causal relationship between gut microbiota and cancers: A two-sample Mendelian randomisation study.. BMC Med..

[9] Yuan Z., Kang Y., Mo C., Huang S., Qin F., Zhang J., Wang F., Jiang J., Yang X., Liang H., Ye L. (2024). Causal relationship between gut microbiota and tuberculosis: A bidirectional two-sample Mendelian randomization analysis.. Respir. Res..

[10] Horai R., Caspi R.R. (2019). Microbiome and autoimmune uveitis.. Front. Immunol..

[11] Zhang Y., Zhou X., Lu Y. (2022). Gut microbiota and derived metabolomic profiling in glaucoma with progressive neurodegeneration.. Front. Cell. Infect. Microbiol..

[12] Beli E., Yan Y., Moldovan L., Vieira C.P., Gao R., Duan Y., Prasad R., Bhatwadekar A., White F.A., Townsend S.D., Chan L., Ryan C.N., Morton D., Moldovan E.G., Chu F.I., Oudit G.Y., Derendorf H., Adorini L., Wang X.X., Evans-Molina C., Mirmira R.G., Boulton M.E., Yoder M.C., Li Q., Levi M., Busik J.V., Grant M.B. (2018). Restructuring of the gut microbiome by intermittent fasting prevents retinopathy and prolongs survival in db/db mice.. Diabetes.

[13] Danca C., Costea C.F., Costan V.V., Turliuc M.D., Sava A., Turliuc S., Cucu A., Dragomir R.A., Sadiye Scripcariu I., Dumitrescu N., Carauleanu A. (2018). Gut microbiota and serotonin - biochemical pathways in age-related macular disease.. Revista de Chimie.

[14] Lincke J.B., Christe L., Unterlauft J.D., Zinkernagel M.S., Zysset-Burri D.C. (2023). Microbiome and retinal vascular diseases.. Am. J. Pathol..

[15] Zinkernagel M.S., Berger L., Herzog E.L., Wolf S., Zysset D. (2023). Compositional and functional features of the gut microbiome in patients with acute onset central serous chorioretinopathy.. Invest. Ophthalmol. Vis. Sci..

[16] Davies N.M., Holmes M.V., Davey Smith G. (2018). Reading Mendelian randomisation studies: A guide, glossary, and checklist for clinicians.. BMJ.

[17] Kurilshikov A., Medina-Gomez C., Bacigalupe R., Radjabzadeh D., Wang J., Demirkan A. (2021). Large-scale association analyses identify host factors influencing human gut microbiome composition.. Nat. Genet..

[18] Au Yeung S.L., Gill D. (2023). Standardizing the reporting of Mendelian randomization studies.. BMC Med..

[19] Liu K., Zou J., Fan H., Hu H., You Z. (2022). Causal effects of gut microbiota on diabetic retinopathy: A Mendelian randomization study.. Front. Immunol..

[20] Jia J., Dou P., Gao M., Kong X., Li C., Liu Z., Huang T. (2019). Assessment of causal direction between gut microbiota–dependent metabolites and cardiometabolic health: A bidirectional mendelian randomization analysis.. Diabetes.

[21] Luo S., Li W., Li Q., Zhang M., Wang X., Wu S., Li Y. (2023). Causal effects of gut microbiota on the risk of periodontitis: A two-sample Mendelian randomization study.. Front. Cell. Infect. Microbiol..

[22] Bowden J., Davey Smith G., Burgess S. (2015). Mendelian randomization with invalid instruments: Effect estimation and bias detection through Egger regression.. Int. J. Epidemiol..

[23] Bowden J., Davey Smith G., Haycock P.C., Burgess S. (2016). Consistent estimation in mendelian randomization with some invalid instruments using a weighted median estimator.. Genet. Epidemiol..

[24] Xiang M., Wang Y., Gao Z., Wang J., Chen Q., Sun Z., Liang J., Xu J. (2023). Exploring causal correlations between inflammatory cytokines and systemic lupus erythematosus: A Mendelian randomization.. Front. Immunol..

[25] Chen X., Kong J., Pan J., Huang K., Zhou W., Diao X., Cai J., Zheng J., Yang X., Xie W., Yu H., Li J., Pei L., Dong W., Qin H., Huang J., Lin T. (2021). Kidney damage causally affects the brain cortical structure: A Mendelian randomization study.. EBioMedicine.

[26] Xiang K., Wang P., Xu Z., Hu Y.Q., He Y.S., Chen Y., Feng Y.T., Yin K.J., Huang J.X., Wang J., Wu Z.D., Yang X.K., Wang D.G., Ye D.Q., Pan H.F. (2021). Causal effects of gut microbiome on systemic lupus erythematosus: A two-sample mendelian randomization study.. Front. Immunol..

[27] Li H., Liu S., Zhang K., Zhu X., Dai J., Lu Y. (2023). Gut microbiome and plasma metabolome alterations in myopic mice.. Front. Microbiol..

[28] Omar W.E.W., Singh G., McBain A.J., Cruickshank F., Radhakrishnan H. (2024). Gut microbiota profiles in myopes and nonmyopes.. Investigative Ophthalmol. Vis. Sci..

