# Supplementary material for: Deciphering the Microbiome-Gut-Eye Axis: A Mendelian Randomization Analysis of the Causal Influence of Gut Microbiota on Myopia
Source: Comb Chem High Throughput Screen. 2025 Apr 29;29(8):1311–7. doi: 10.2174/0113862073385717250415110224 (PMC13539582; doi:10.2174/0113862073385717250415110224)
Supplement: Supplementary file 1 [file CCHTS-29-8-1311_SD1.pdf]

Supplementary Material

Deciphering the Microbiome-Gut-Eye Axis: A Mendelian Randomization Analysis of the Causal Influence of Gut Microbiota on Myopia

Weicheng Xu[1,2] and Wei Shi[1,3,*]

[1]*First Clinical Medical College, Nanjing University of Chinese Medicine, Nanjing, Jiangsu, China;* [2]*Department of Ophthalmology, Rugao Affiliated Hospital of Nanjing University of Chinese Medicine, Nantong, Jiangsu, China;* [3]*Department of Ophthalmology, Jiangsu Province Hospital of Chinese Medicine, Nanjing, Jiangsu, China*

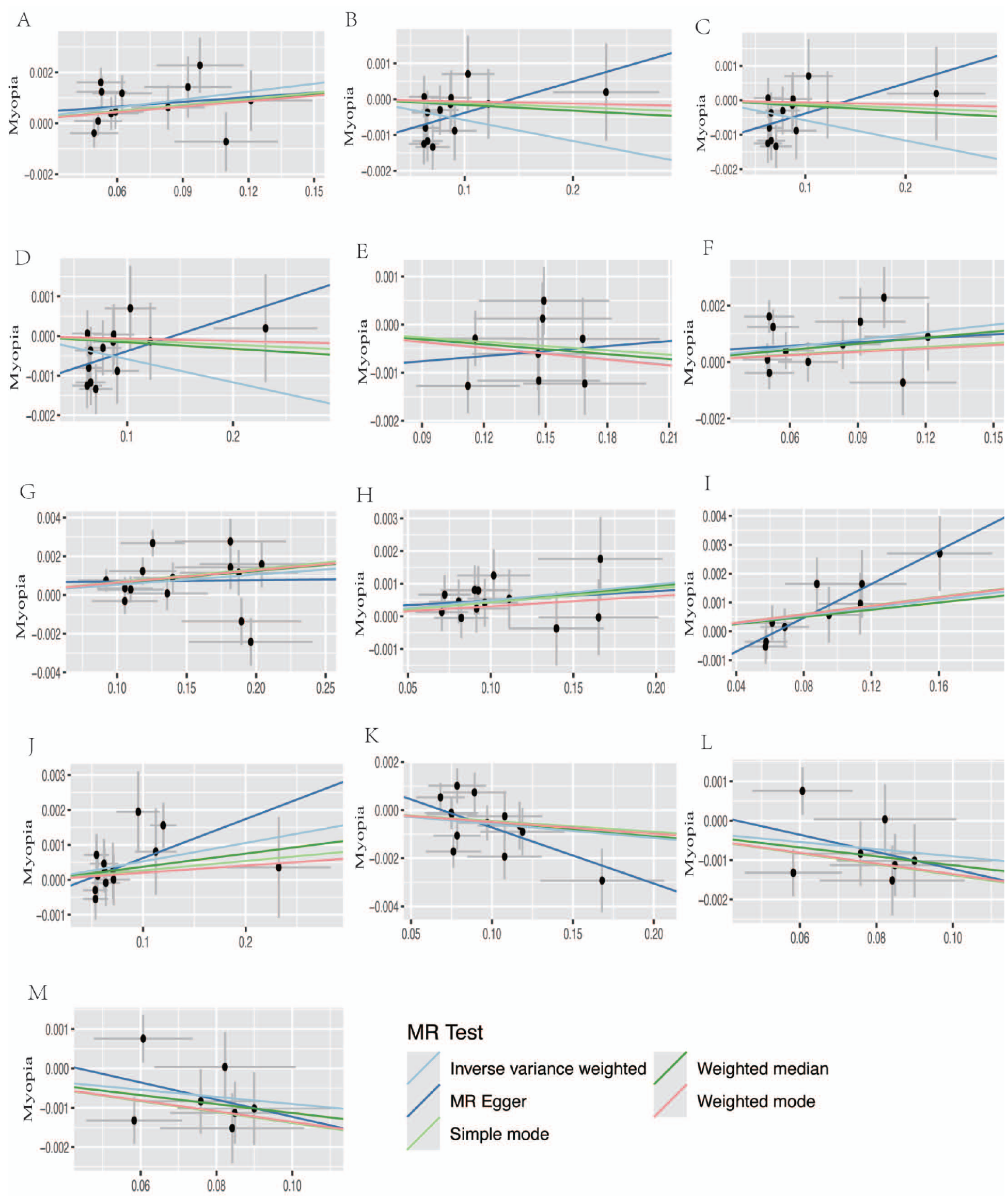

S1 Fig. Scatter plots of the causal effect of GM on myopia. (IVW *P*<0.05). (A)class *Betaproteobacteria* id.2867 .(B)order *Mollicutes RF9* id.11579.(C) unknown family id.1000005471.(D) unknown genus id.1000005472.(E) genus *Allisonella* id.2174.(F) order *Burkholderiales* id.2874.(G) family *Oxalobacteraceae* id.2966.(H) unknown genus id.2755.(I) genus *Eubacterium xylanophilum group* id.14375.(J) genus *Bifidobacterium* id.436.(K) genus *Lachnospiraceae UCG001* id.11321.(L) family *Enterobacteriaceae* id.3469.(M) order *Enterobacteriales* id.3468.

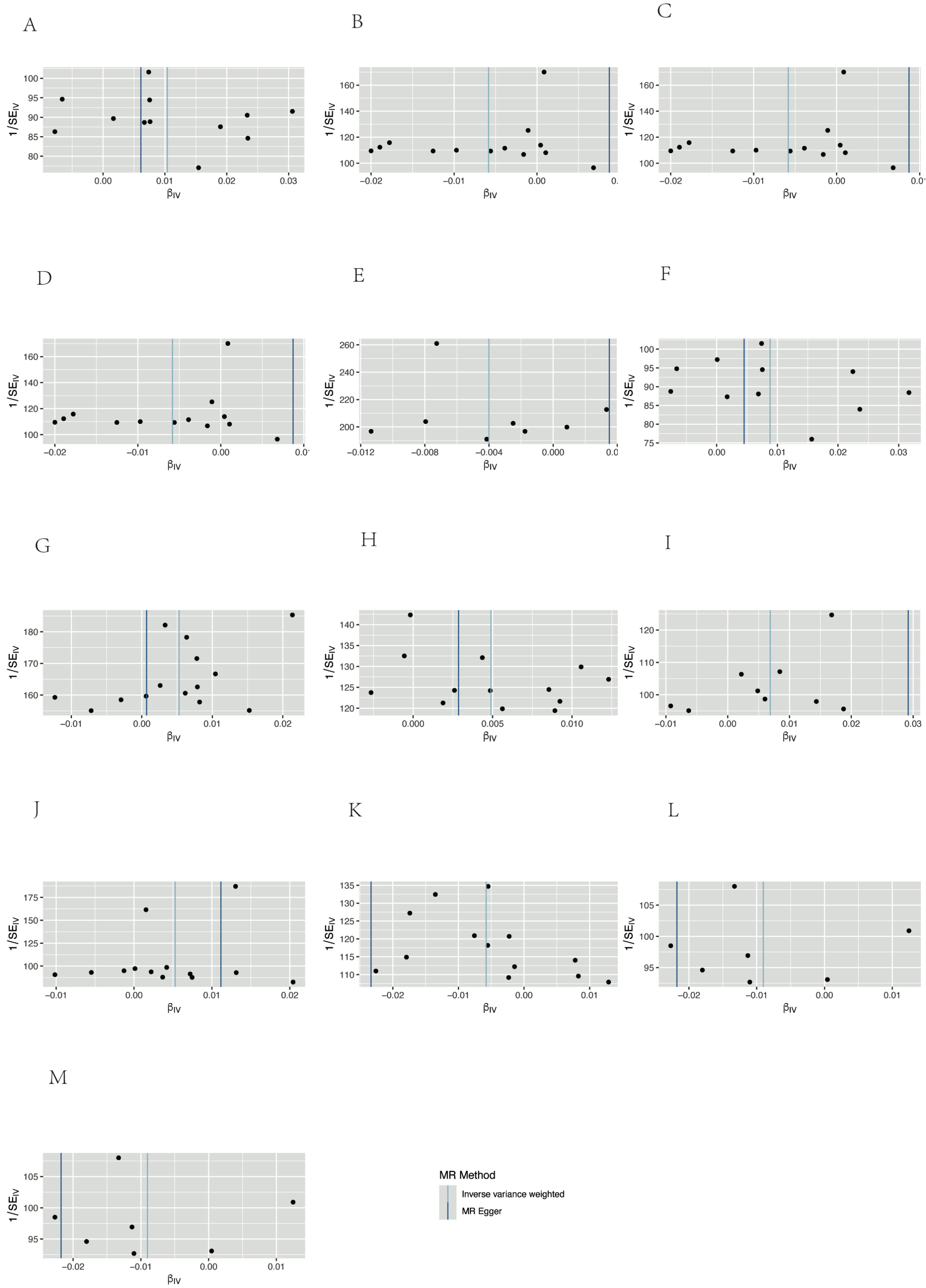

S2 Fig. Funnel plots of the MR estimates for the significant causality of 13 GM taxa and the risk of myopia. (A)class *Betaproteobacteria* id.2867 .(B)order *Mollicutes RF9* id.11579.(C) unknown family id.1000005471.(D) unknown genus id.1000005472.(E) genus *Allisonella* id.2174.(F) order *Burkholderiales* id.2874.(G) family *Oxalobacteraceae* id.2966.(H) unknown genus id.2755.(I) genus *Eubacterium xylanophilum group* id.14375.(J) genus *Bifidobacterium* id.436.(K) genus *Lachnospiraceae UCG001* id.11321.(L) family *Enterobacteriaceae* id.3469.(M) order *Enterobacteriales* id.3468.

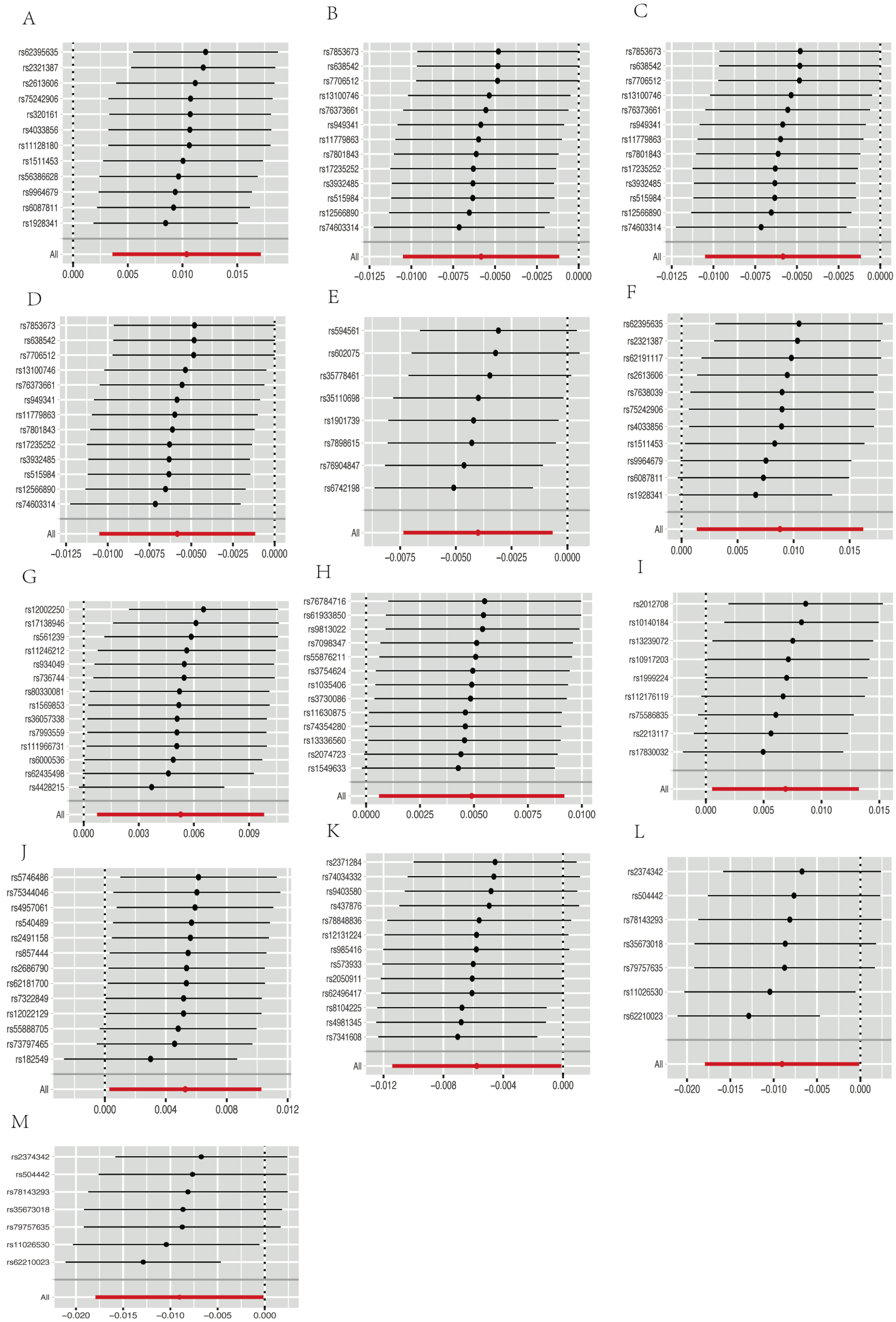

S3 Fig. MR leave-one-out sensitivity analysis for GM on myopia. (A)class *Betaproteobacteria* id.2867 .(B)order *Mollicutes RF9* id.11579.(C) unknown family id.1000005471.(D) unknown genus id.1000005472.(E) genus *Allisonella* id.2174.(F) order *Burkholderiales* id.2874.(G) family *Oxalobacteraceae* id.2966.(H) unknown genus id.2755.(I) genus *Eubacterium xylanophilum group* id.14375.(J) genus *Bifidobacterium* id.436.(K) genus *Lachnospiraceae UCG001* id.11321.(L) family *Enterobacteriaceae* id.3469.(M) order *Enterobacteriales* id.3468.
